# Antibiotic resistance patterns in human, animal, food and environmental isolates in Ghana: a review

**DOI:** 10.11604/pamj.2020.35.37.18323

**Published:** 2020-02-12

**Authors:** Pilar García-Vello, Bruno González-Zorn, Courage Kosi Setsoafia Saba

**Affiliations:** 1 Personal Support for Projects of Molecular Biology and Biotechnology, Faculty of Pharmacy, Complutense University of Madrid, Madrid, Spain; 2 Animal Health Department, Faculty of Veterinary, Complutense University of Madrid, Madrid, Spain; 3 Biotechnology Department, Faculty of Agriculture, University for Development Studies, Tamale, Ghana

**Keywords:** Infections, antibiotic resistance, public health, Ghana

## Abstract

Many articles have been published on resistant microorganisms isolated from humans, animals, foods and the environment in Ghana. However, there are no reviews that summarize the information on the isolates and antibiotics tested so far in the country. This literature review was completed through “PubMed” and “Google Scholar” searches. We included publications from the period 1975-2015 with a laboratory-based methodology to determine antibiotic resistance of strains isolated in Ghana. In total, 60 articles were included in the analysis with 10% of the articles carrying out nationwide research on antibiotic resistance. The regions of Ghana with the highest published articles were Greater Accra (40%), Ashanti (21.7%) and Northern Region (10%). Most of the studies (86.7%) were related to isolates collected from human samples followed by environmental (5%), animal (3%) and food samples (2%). Ten different bacteria genera were observed in the studies. The most common was *Escherichia coli*, followed by *Staphylococcus* spp., *Mycobacterium* spp. and *Streptococcus* spp. The highest mean resistance rate was encountered in *Escherichia coli* (62.2%) followed by *Klebsiella* spp*.* (60.4%) and *Pseudomonas* spp. (52.1%). High resistance rates have been found in Ghana, however, the data are skewed and some regions of the country have been neglected. There is a need for higher quality research to establish and monitor resistance patterns in Upper West, Brong-Ahafo, Volta and Eastern Regions of Ghana.

## Introduction

The increasing problem of antibiotic resistance remains a major global health problem and causes a huge medical burden [1]. There are significant international efforts, notably the World Health Organization (WHO) and Food and Agriculture Organizations (FAO), working towards tackling this crisis through planned programs such as antibiotic stewardship programs coordinated by the Global Action Plan on Antimicrobial Resistance of the WHO [2]. These programs promote optimal antimicrobial use [3-6], resistance surveillance [7], research into the molecular bases for resistance [8], strict regulations on antibiotic usage [9-11] and education of medical, public health officials and the general populace [12-14]. Some private organizations and individuals have made efforts to tackle the antibiotic crisis in some African countries. An example can be seen in Ghana through the sponsorship of research projects and educational programs [15-22]. Unfortunately, some of these projects may be duplicated and have narrow coverage area due to limited funding. Others may be executed successfully but their impacts are minimal. Furthermore, complex socio-economic and political conditions in developing and low-income countries [23] make antibiotic resistance a difficult problem to tackle [24]. This is further hindered by the inadequate and unpublishable data produced [25]. A systematic review of publications of these research works is important to evaluate the overall impacts of antibiotics on human, animal, food and environmental isolates. The objective of this study was to compile all the available information concerning antibiotic resistance in Ghana to assist clinicians and researchers to know the most commonly isolated microbes and their resistance patterns. This research aims to provide further information on the current antibiotic resistance problems, which may also help institutions to create programs and policies to enhance antimicrobial stewardship, reduce misuse of antibiotics by health professionals and unskilled practitioners, control drug quality, limit the spread of resistant bacteria, develop adequate surveillance systems and update the Standard Treatment Guidelines of hospitals in Ghana [26]. These will help to prevent or slow the emergence of resistance among microorganisms. Furthermore, this review aims to inspire researchers to conduct more investigations, particularly in neglected areas of the country.

## Methods

A thorough search of literature was done in Medline (PubMed) and Google Scholar electronic databases. The search was carried out in June 2015. The following keywords were used to search in both databases with no language restriction or methodological filters: Antimicrobial OR Antibiotic AND resistance AND Ghana. Abstracts of potentially relevant titles were then reviewed manually for eligibility and were selected for closer examination to be included in the study.

**Search on PubMed**: the articles from PubMed included all publications concerning antibiotics resistance in Ghana from 1975 to July 2015.

**Search on Google Scholar**: the same search words were used on Google Scholar, producing over ten thousand results. Consequently, a selection was done of every 10^th^ page (pages 1, 10, 20, 30, 40, 50) and articles related to antibiotics in Ghana were selected based on their titles and abstracts.

**Inclusion and exclusion criteria**: the inclusion criteria were: (i) abstract availability, (ii) information about antibiotic resistance of at least one bacterium, (iii) laboratory-based methodology, (iv) bacterial strains collected in Ghana. The exclusion criteria were: (i) articles already included from PubMed but found on Google Scholar (ii) articles without abstract (iii) abstracts without factual data on antibiotic resistance.

**Data analysis**: data were gathered from each article concerning the bacteria isolated, the origin of isolation (human, animal, food and environment), the methods of antibiotic susceptibility test and the antibiotics used for them, the most frequent antibiotics used, the most resistant bacteria to the antibiotics used. The particular regions where the researches were conducted were also recorded. Data compilation and analysis were performed using the software MS Excel^®^ and results were presented in percentages using tables and figures.

## Current status of knowledge

**Figure 1:**
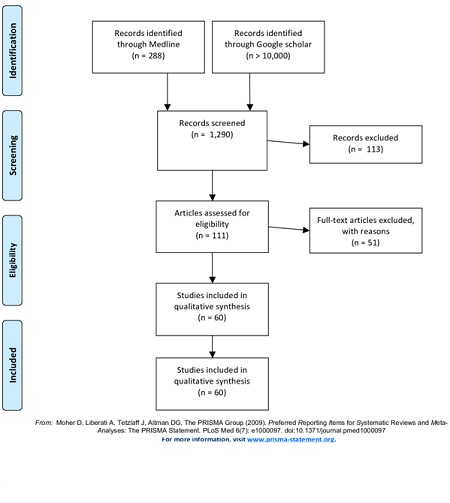
Selection of records for the analysis

**Current status of knowledge**: the search on PubMed resulted in 288 publications of which 188 came from the search word “Antimicrobial AND resistance AND Ghana” and 100 for “Antibiotic AND resistance AND Ghana”. Over ten thousand results were obtained using Google Scholar. After the screening against the inclusion criteria, only 60 articles were included in the analysis (Figure 1). Of the 60 articles analysed, 20 articles were not available in full hence only the abstracts were reviewed [27-46].

### Sample collection

**Figure 2:**
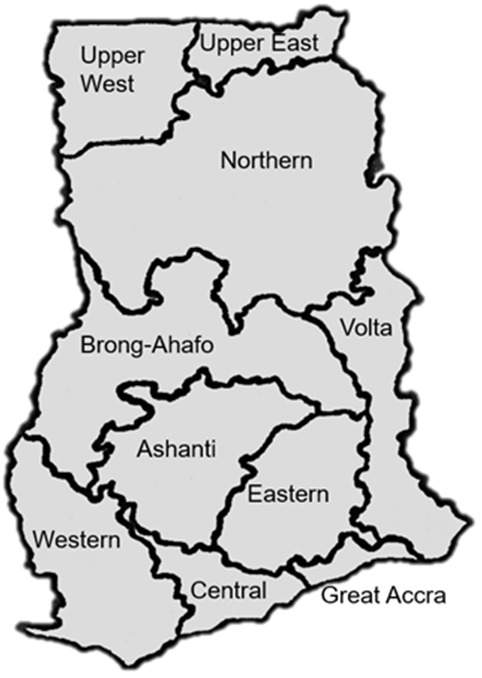
Ghana map

**Figure 3:**
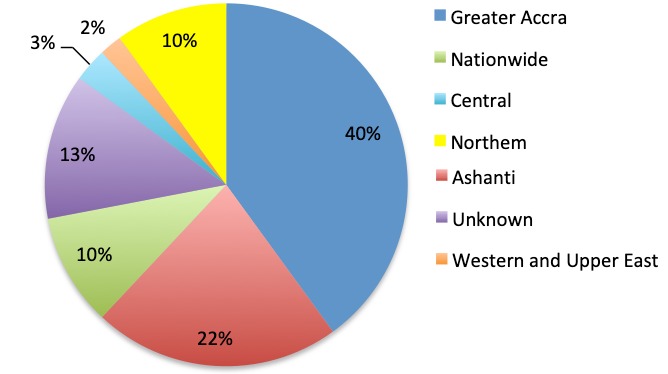
Geographical distribution of the studies

**Geographical/regional distribution of articles**: Ghana has ten different administrative regions (Figure 2). Research on antibiotics resistance was not conducted in the Upper West Region, Brong Ahafo Region, Volta Region and Eastern Region per this review as of the time of data collection. The majority, 23(38.3%) of the articles came from the Greater Accra Region [29,34,42,46,47-65] of which the capital city of Ghana, Accra is located (Figure 3). Six (10.0%) of the 60 articles carried out nationwide research on antibiotic resistance [43,66-70], thus these studies were designed to take samples from the whole of Ghana. Eight (13.3%) of the articles could not be traced due to inadequate information on the locations of the research [27,30,31,33,38,41,71,72].

**Figure 4:**
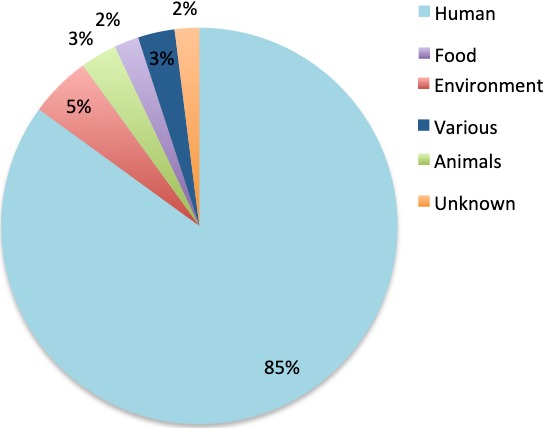
Type of samples included in the studies

**Types of samples collected**: the 60 articles analysed obtained samples from different sources (Figure 4). The human isolates were taken from both healthy individuals and patients with previous or no treatment. Samples were mostly blood (8 articles) [34,41,46,54,68,73-75], and nasopharyngeal and nasal samples (6) [32,37,49,52,61,66]. There were also faecal (5) [53,59,63,71,76], sputum (3) [39,70,77], cerebrospinal fluid (3) [29,78,79], urine (3) [33,47,54], skin (1 diabetic foot ulcers and 1 Buruli ulcer) [80,65], bile (1) [30] and vaginal, cervix and urethra samples (1) [28]. Nine articles obtained samples from different sources and analysed the results together [40,51,56,57,60,69,81,82]. Ten of the articles described the kind of patient as adult or child but did not describe the origin of the samples [31,35,38,43-45,58,62,67,83]. From the three environmental articles, samples were collected from beds, floors and drainage systems of hospitals (1) [84], water samples (1) [72] and mobile phones (1) [85]. Additionally, one research group exclusively sampled tiger nuts [48]. The articles that studied antibiotic resistance in animals sampled from domesticated animals (rectal and cloacae swabs) [27] and hospital cockroaches [64]. There were two articles with combined sample sources; one collected human and animal samples; faecal samples from farmers and animals from farms [50]. Another investigated animal and food sources; samples were taken from poultry in three farms and carcasses from two open markets and a cold store [42].

**Genus and species of bacteria isolated**: the bacterial species isolated and identified in the sixty articles were from ten different genera: *Escherichia coli, Staphylococcus* spp., *Streptococcus* spp., *Klebsiella* spp., *Salmonella* spp., *Pseudomonas* spp., *Mycobacterium* spp., *Neisseria* spp., *Proteus* spp. and *Shigella* spp. More information about the isolation of each genus of bacteria can be found in Table 1.

**Table 1:** Isolation, antibiotic susceptibility test and focus of the study

Bacterium	Isolation	Test ^a^	Exclusive ^b^
***Escherichia coli*** (17 articles) [30,40,42,47,50,51,58,59,64,69,71,73,74,80,81,84,85]	Humans (13) [30,40,47,50,51,58,59,69,71,73,74,80,81,84] Humans and animals (1) [50] Animals (1) [64] Food and animals (1) [42] Environment (1) [85]	Disk diffusion (11) [42,47,50,51,58,59,64,73,74,80,85] Dilution (2) [71,84] Vitex 2 bioMerieux (1) [81] E-test (1) [69] Unknown (2) [30,40]	(5) [50,59,71,81,85]
***Staphylococcus* spp.** (14 articles) [34,35,40,47-49,51,52,55,65,68,69,73,74]	Humans (12) [34,35,40,47,49,51,52,55,65,68,69,73,74] Food (tiger nuts) (1)[48]	Disk diffusion (11) [35,47,48,49,51,52,65,68,69,73,74] Disk diffusion + E-test (1) [55] Unknown (2) [34,40]	(1) [34]
***Salmonella* spp.** (9 articles) [30,42,46,54,56,57,68,75,76]	Humans (8) [30,46,54,56,57,68,75,76] Animals and food (1) [42]	Disk diffusion (6) [54,56,57,68,75,76] Unknown (3) [30,42,46]	All (9) [30,42,46,54,56,57,68,75,76]
***Streptococcus* spp.** (11 articles) [29,31,32,35,40,49,61,66,67,75,79]	Humans (11) [29,31,32,35,40,49,61,66,67,75,79]	Disk diffusion (5) [31,49,66,67,75] E-test (1) [79]. Unknown (5) [29,32,35,40,61]	(6) [31,32,61,66,67,79]
***Mycobacterium* spp.** (6 articles) [36,37,39,44,70,77]	Humans (6) [36,37,39,44,70,77]	Proportion method (3) [39,70,77] Unknown (3) [36,37,44]	All (6) [36,37,39,44,70,77]
***Neisseria* spp.** (6 articles) [28,29,33,43,45,78]	Humans (6) [28,29,33,43,45,78]	E-test (1) [78] Unknown (5) [28,29,33,43,45]	All (6) [36,37,39,44,70,77]
***Pseudomonas* spp.** (7 articles) [30,51,65,68,69,80,83]	Humans (7) [30,51,65,68,69,80,83]	Disk diffusion (5) [51,65,68,80,83] Disk diffusion + E-test (1)[69] Unknown (1) [30]	None
***Proteus* spp.** (6 articles) [48,51,65,80,82,83]	Humans (5) [51,65,80,82,83] Food (tiger nuts) [48]	Disk diffusion (6) [48,51,65,80,82,83]	(1) [82]
***Klebsiella* spp.** (10 articles) [30,48,51,60,64,69,73,74,80,86]	Humans (7) [30,51,60,69,73,74,80] Animals (1) [64] Food (1) [48] Environment (1) [86]	Disk diffusion (8) [30,48,51,64,73,74,80,86] Disk diffusion + E-test (1)[69] Disk diffusion + dilution (1) [60]	None
***Shigella* spp.** *Shigella* spp. (3 articles) [42,63,84]	Humans (2) [63,84] Animals and food (1) [42]	Disk diffusion method +E-test (1) [63] Breakpoint microdilution (1) [84] Unknown (1) [42]	(1) [63]

This table has been elaborated using the general data from bacteria, with no discrimination of region or sample origin

a Methodology used to test the susceptibility of bacteria to antibiotic

b Exclusive: studies that were conducted exclusively on this bacterium, without including other genera

**Antibiotic resistance patterns**: the information on the most tested antibiotics and the antibiotics recording the highest resistance rates was compiled, producing a table with forty antibiotics and 3 antibiotic combinations (Table 2). The resistance level of all the organisms isolated and their resistance patterns to forty-three antibiotic formulations and the number of bacteria tested were also included in the table. Weighted averages were calculated to obtain data that allows a comparison between bacteria. The highest resistance was found in *E. coli* (62.2%) followed by *Klebsiella* spp. (60.4%) and *Pseudomonas* spp. (52.1%) (Table 2).

**Table 2:** Summary of resistance rates findings

Antibiotic	*E. coli*	Staph.	Strept.	Klebs.	Salmo.	Mycob.	Pseudo.	Neiss.	Prote.	Shige.
Amikacin	40.19	0	-	18.5	0	-	5.3	-	5.45	37.5
Aminopenicillin	-	-	-	-	84.5	-	-	-	-	-
Amoxicillin	100	75	-	-	-	-	-	-	-	-
Ampicillin	87.14	86.9	66.8	55.8	93.5	-	100	0	81	95.8
Cefadroxil	100	-	-	-	100	-	-	-	-	-
Cefotaxime	8.76	12.5	2.4	65.5	0	-	45.5	0	38.2	12.5
Cefotiam	100	-	-	-	100	-	-	-	-	-
Ceftazidime	13.3	-	-	80	0	-	7.8	-	29.8	-
Ceftriaxone	26.9	-	4.19	20	0	-	52	R	41.4	-
Cefuroxime	50.3	60.9	R	33.3	32	-	78.8	-	51.4	16.7
Chloramphenicol	49.1	45.9	R	73.9	R	-	84.6	0	90.4	R
Ciprofloxacin	19.4	15.8	2.8	20.3	0	0	11.4	R	35.4	0
Cloxacillin	100	4.7	0	100	-	-	-	-	-	-
Colistin	-	-	-	-	0	-	-	100	-	-
Cotrimoxazole	81.2	54.5	100	82.4	76.5	-	81.6	R	84.2	91.7
Ethambutol	-	-	-	-	-	0.24	-	-	-	-
Erythromycin	100	7.3	12.67	100	100	-	-	R	-	-
Fluoroquinolone	9.9	-	-	-	-	-	-	-	-	0
Fosfomycin	0	-	-	-	-	-	-	-	-	0
Gentamicin	26.1	R	77.8	29.7	16.9	-	20.57	-	25.6	37.5
Isoniazid	-	-	-	-	-	20.6	-	-	-	-
Methicillin	-	63.8	-	-	-	-	-	-	-	-
Mezlocillin	76	-	-	-	-	-	-	-	-	-
Nalidixic acid	32.5	-	-	-	0	-	-	0	-	0
Ofloxacin	29.6	0	-	44.7	-	-	-	-	0	0
Oleandomycin	-	-	-	-	-	-	-	4.9	-	-
Oxacillin	-	4.9	15.1	-	-	-	-	-	-	-
Oxitetracycline	90	-	-	-	-	-	-	-	-	-
Penicillin	100	90.2	R	100	100	-	-	R	-	-
Pyrazinamide	-	-	-	-	-	1.5	-	-	-	-
Rifampicin	-	-	-	-	-	3.2	-	0	-	-
Septrim	-	-	-	-	-	-	-	13	-	-
Streptomycin	70	-	-	-	0	18.2	-	38.8	-	-
Sulfamethoxazole	-	-	33.9	-	0	-	-	-	-	-
Sulphadiazine	-	-	-	-	-	-	-	41.86	-	-
Sulphafiurazole	-	-	-	-	-	-	-	8.1	-	-
Sulphonamide	70	-	-	60	-	-	-	-	-	-
Tetracycline	74.9	41.9	R	82.6	10.7	-	85.4	R	82.5	91.7
Thiacetazone	-	-	-	-	-	4.08	-	-	-	-
Trimethoprim	74.2	67	99.1	-	0	-	-		-	-
Amox-Ampi	83.3	-	20	-	-	-	-	-	-	R
Amoxiclav	52.6	29.3	11.1	-	72.1	-	-	-	-	-
Trimeto-Sulf	76	0	R	-	89.5	-	-	-	-	R
Total resistance	62.2	36.7	34.3	60.4	39.8	6.8	52.1	18.8	47.1	31.9

Resistance rates are expressed in percentages (%)

**R**: some of the resistances were not included in the articles as a numeric value but as resistant or not resistant. The cases where bacteria were resistant to antibiotics but the rate is unknown have been written as an R.

Ecoli: *Escherichia coli;* Staph.: *Staphylococcus* spp.; Strept.: *Streptococcus* spp.; Klebs.: *Klebsiella* spp. ; Salmo: *Salmonella* spp.; Mycob: *Mycobacterium* spp.; Pseudo: *Pseudomonas* spp.; Neiss: *Neisseria* spp.; Prote: *Proteus* spp.; Shige: *Shigella* spp.;

This table has been elaborated using the general data from bacteria, with no discrimination of region or sample origin.

Antibiotics not tested in a bacteria were not included in the calculations of the total resistance of that bacteria

**Antibiotic resistance pattern of *E. coli***: the general resistance rates from human isolates were: 100% to Amoxicillin [42,47], 86.8% to Ampicillin [40,42,50,58,59,64,71,74,80,81,84,85], 81.6% to Cotrimoxazole [40,50,59,64,73,74,80,81,85], 73.6% to Tetracycline [42,58,59,64,74,80,84], 48.7% to Chloramphenicol [42,50,58,64,71,73,80,81,84], 34.42% to Cefuroxime [50,59,74,80,81,84], 22.6% to Gentamicin [40,47,50,59,64,71,73,74,80,81,84,85], 12.74% to Amikacin [50,59,64,73,74], 6.97% to Cefotaxime [50,59,64,80] and 5.7% to Ciprofloxacin [58,59,64,71,73,74,80,84,85]. The total resistance rates in animals were: 95% to Cefuroxime, 83.4% to Tetracycline, 66% to Cefotaxime, 62% to Chloramphenicol, 60.5% to Gentamicin, 56.2% to Amikacin, 50% to Cotrimoxazole, 25.4% to Ampicillin and 20% to Ciprofloxacin [42,50,64]. In the environmental samples, antibiotic resistance rates were: 90.7%, to Ampicillin, 78.4% to Cotrimoxazole, 46.4% to Gentamicin, and 32 % to Ciprofloxacin [85]. All the *E. coli* isolates tested were resistant (100%) to Tetracycline in Ashanti Region [80], Cefadroxil and Cefotiam in Accra [42], Cloxacillin and Erythromycin in Northern Region [74] and Penicillin in Accra and Northern Region [42,74].

**Antibiotic resistance pattern of *Staphylococcus* spp.**: the resistance rates in human isolates were: 94.1% to Ampicillin [49,65,74], 90.2% to Penicillin [40,49,55,65,73], 60.9% to Cefuroxime [49,65,74], 63.8% to Methicillin [55,65,73], 59.9% to Cotrimoxazole [49,65,73,74], 40.4% to Tetracycline [55,65,74], 22.8% to Ciprofloxacin [73,74] and 7.3% to Erythromycin [49,55,65,73,74]. Antibiotic resistance rates in food were: 0% to Gentamicin, 12.5% to Ampicillin, 0% to Cotrimoxazole, 82.5% to Tetracycline and 0% to Ciprofloxacin [48]. All isolates from the Northern Region were resistant to Cotrimoxazole and Ampicillin [74]. In addition, the resistance was very high against Penicillin (92.4%), Flucoxacillin (83.3%), Ampicillin (82.8%), Amoxicillin (75.0%), Methicillin (72.0%), Amoxicillin (62.5%) and Cefuroxime (62.2%) in Accra [34,47,48,49,51,55,65]; Penicillin (90.0%) and Cotrimoxazole (66.0%) in Ashanti Region [40,73]; Ciprofloxacin (66.7%) and Tetracycline (66.7%) in Northern Region [74]. There were no animal isolates.

**Antibiotic resistance pattern of *Salmonella* spp.**: the resistance rates in human isolates were: 92.3% to Ampicillin [54,68,76], 83.2% to Chloramphenicol [54,68,75,76], 72.1% to Amoxicillin-Clavulanic acid [54,68,75,76], 76.5% to Cotrimoxazole [54,75], 32% to Cefuroxime [54,68,75], 16.9% to Gentamicin [54,68,75,76], 10.7% to Tetracycline [54,68,75,76], 0% to Ciprofloxacin [54,68,75,76]. There were no quantified resistance to Amikacin [54,76], Cefotaxime [54,76] and Nalixilic acid [76]. The resistance rates in animals and foods were also not clearly specified [42]. The highest resistance was found in Accra, where all tested bacteria were resistant to Ampicillin, Cefadroxil, Cefotiam, Erythromycin and Penicillin [42,46,54,56,57], followed by Ashanti Region where the resistance was also high against Aminopenicillin (84.5%), Cefuroxime (53.5%), Chloramphenicol (81.9%), Cotrimoxazole (76.5%) and the combination Amoxicillin-Clavulanic acid (74.3%) [75].

**Antibiotic resistance pattern of *Streptococcus* spp.**: the resistance rates in human isolates were: 100% to Cotrimoxazole [35,49,61,75], 66.84% to Ampicillin [49,61], 12.67% to Erythromycin [32,49,61], 4.19% to Ceftriaxone [32,61,75] and 2.38% to Cefotaxime [32]. There were resistances to Penicillin [32,35,40,49,61], Tetracycline [61,75], Chloramphenicol [32,40,65] and Cefuroxime [32,49,65] but the exact percentages were not specified. There were no animal isolates. The highest resistance registered was Cotrimoxazole (100%) in Accra and Ashanti Regions [35,49,61,75]. In addition, high resistance rates found for Ampicillin (67.2%), Penicillin (48.1%), Erythromycin (20.9%) and Tetracycline (78.0%) in Accra [29,49,61]; and Gentamicin (77.8%), Ciprofloxacin (47.4%), Chloramphenicol (20.5%) and Tetracycline (75.0%) in Ashanti Region [32,35,40,75].

**Antibiotic resistance pattern of *Mycobacterium* spp.**: the antibiotics tested in the different publications were: Isoniazid (6 articles) [36,37,39,44,70,77], Ethambutol (4) [37,44,70,77], Pyrazinamide (3) [37,44,70], Rifampicin (3) [37,44,77], Streptomycin (4) [37,44,77], Thioacetazone (1) [44], and Ciprofloxacin (1) [37]. The resistance rates in human isolates were: 20.6% to Isoniazid [36,37,39,44,70,77], 18.17% to Streptomycin [37,44,77], 4.08% to Thioacetazone [44], 3.22% to Rifampicin [37,44,77], 1.54% to Pyrazinamide [37,44,70], 0.24% to Ethambutol [37,44,70,77], and 0% to Ciprofloxacin [37]. The highest resistance was found against Streptomycin in Central Region (33%) [77]; followed by the resistance in Ashanti Region against Thioacetazone (29%), Isoniazid (26.2%) and Streptomycin (18.4%) [37,44] and in Central Region against Isoniazid (13.4%), Rifampicin (6.7%) and Ethambutol (4.1%) [77]. No animal isolates were obtained.

**Antibiotic resistance pattern of *Neisseria* spp.**: resistance rates in human isolates were: 100% to Colistin [45], 41.86% to Sulphadiazine [45,78], 38.8% to Streptomycin [45], 13% to Septrim [45], 8.1% to Sulphafurazole [45,78], and 4.9% to Oleandomycin [45]. There was also resistance to Penicillin [28,33,43,45,78], Tetracycline [28,33,43,45,78], Ciprofloxacin [33,43,78], Chloramphenicol [45,78], and Erythromycin but were not quantified [28,45]. No animal isolates were obtained. The highest resistance rates were found in Upper East Region [78] and Northern Region [45]. In Upper East Region, all the Neisseria species tested were resistant to Sulphadiazine [78]. In the Northern Region, all the Neisseria species tested were resistant to Colistin, followed by Streptomycin (38.8%), Septrim (13.0%), Sulphafurazole (8.1%), Sulphadiazine (8.1%) and Oleandomycin (4.9%) [45]. The bacteria from other regions were susceptible to all antibiotics tested, except Ashanti Region. The data from Ashanti Region were not quantitative but instead labelled as “R” signifying resistance against Ceftriaxone, Cotrimoxazole, Erythromycin, Norfloxacin, Penicillin, Spectinomycin and Tetracycline [28].

**Antibiotic resistance pattern of *Pseudomonas* spp.**: the most tested antibiotics were Cefotaxime (4 articles) [51,65,80,83], Cefuroxime (4) [51,65,80,83], Ceftriaxone (3) [65,80,83], Gentamicin (3) [65,80,83], Ampicillin (3) [51,65,80], Chloramphenicol (3) [51,65,80], Cotrimoxazole (3) [51,65,80], Tetracycline (3) [51,65,80], Ceftazidime (2) [80,83] and Ciprofloxacin (2) [80,83], and were also the antibiotics that recorded the highest resistance rates. Resistance rates in human isolates were: 45.5% to Cefotaxime [51,65,80,83], 52% to Ceftriaxone [65,80,83], 78.8% to Cefuroxime, [51,65,80,83], 20.57% to Gentamicin [65,80,83], 100% to Ampicillin [51,65,80], 7.8% to Ceftazidime [80,83], 84.6% to Chloramphenicol [65,80], 11.4% to Ciprofloxacin [80,83], 81.6% to Cotrimoxazole [51,65,80] and 85.38% to Tetracycline [51,65,80]. No animal isolates were obtained. The highest resistance rates were found in Accra and Ashanti Regions. In Accra, the resistance was 100% to Ampicillin and Cefuroxime [51]. In the Ashanti Region, the resistance was 100% to Ampicillin, Cotrimoxazole, Tetracycline and Chloramphenicol [80,83]. Other high resistance rates were recorded for Cefotaxime (90%), Tetracycline (81.9%), Chloramphenicol (81%) and Cotrimoxazole (77.3%) in Accra [51]; and Cefuroxime (76.5%) and Ceftriaxone (55.2%) in Ashanti Region [80,83].

**Antibiotic resistance pattern of *Proteus* spp.**: all of the 6 articles used the disk diffusion methods. Resistance rates in human isolates were: 5.48% to Amikacin [82,83], 80.97% to Ampicillin [80,82,83], 37.9% to Cefotaxime [80,82,83], 41.4% to Ceftriaxone [80,82,83], 51.4% to Cefuroxime [80,82,83], 90.3% to Chloramphenicol [80,82,83], 35.9% to Ciprofloxacin [80,83], 84.2% to Cotrimoxazole [80,82,83], 25.7% to Gentamicin [80,82,83] and 82.4% to Tetracycline [80,82]. The bacteria isolated from food were resistant to Ampicillin, Cefotaxime, Chloramphenicol, Cotrimoxazole and Tetracycline, but susceptible to Amikacin, Ciprofloxacin and Gentamicin [48]. The highest resistance rates were found in Accra with 100% resistance to Ampicillin, Cefotaxime, Chloramphenicol, Cotrimoxazole and Tetracycline [48,51]. In the Ashanti Region, the isolates were resistant to Chloramphenicol (90.3%), Cotrimoxazole (84.2%), Tetracycline (82.4%), Ampicillin (81.6%) and Cefotaxime (37.1%) [80,82,83].

**Antibiotic resistance pattern of *Klebsiella* spp.**: the resistance rates in human isolates were: 17.9% to Amikacin [60,73,74], 100% to Ampicillin [74,80], 20% to Cefotaxime [80], 85% to Chloramphenicol [73,80], 17.9% to Ciprofloxacin [60,73,80], 82.4% to Cotrimoxazole [73,74,80], 34.7% to Gentamicin [73,74,80] and 100% to Tetracycline [74,80]. The resistance rates in animal isolates were: 25% to Amikacin, 50% to Ampicillin, 75% to Cefotaxime, 75% to Chloramphenicol, 52% to Ciprofloxacin, 88% to Cotrimoxazole, 31% to Gentamicin and 81% to Tetracycline [64]. In food samples, isolates recorded the following resistance rates: 0% to Amikacin, 33.3% to Ampicillin, 33.3% to Cefotaxime, 33.3% to Chloramphenicol, 66.7% to Cotrimoxazole and 66.7% to Tetracycline [48]. The bacteria isolated from the environment did not have any data specifying antibiotic resistance [86]. The highest resistance rates were found in the Ashanti Region [73,80] and the Northern Region [74]. In the Ashanti Region, 100% resistance was found against the combination of Amoxicillin-Ampicillin and Ampicillin and Tetracycline [73,80]. In Northern Region, the highest resistance rates were 100% to Ampicillin, Cotrimoxazole, Erythromycin, Penicillin and Tetracycline [74]. Also, high resistance rates were found against Cotrimoxazole (86.3%), Tetracycline (79.8%), Cefotaxime (71.2%) and Chloramphenicol (71.6%) in the Greater Accra Region [48,51,60,64].

**Antibiotic resistance pattern of *Shigella* spp.**: resistance rates in human isolates were: 37.5% to Amikacin, 95.8% to Ampicillin, 12.5% to Cefotaxime, 16.7% to Cefuroxime, 83.3% to Chloramphenicol, 0% to Ciprofloxacin, 91.7% to Cotrimoxazole, 0% to Fluoroquinolone, 37.5% to Gentamicin, 91.7% to Tetracycline, 0% to Nalixilic acid and 0% for Oxafloxacin. There was resistance to Amoxicillin-Ampicillin and Trimethoprim-Sulfamethoxazole but was not quantified [63]. The bacteria from food were susceptible to Chloramphenicol and Fosfomycin, but the resistance percentages were not specified [42]. The highest resistance rates were found in the Greater Accra Region: Ampicillin (95.8%), Cotrimoxazole (91.7%), Tetracycline (91.7%), Chloramphenicol (66.6%), Amikacin (37.5%), Gentamicin (37.5%), Cefuroxime (16.7%) and Cefotaxime (12.5%) [42].

Antibiotic resistance is one of the biggest threats to international health nowadays; its influence goes beyond health, impacting food security and the holistic development of Ghana. Substantial evidence worldwide indicates that there has been significant increase in antibiotic resistance. Furthermore, high antibiotic resistance rates were found in different bacterial isolates in Ghana [1]. As this study observed, there were numerous gaps in the knowledge about resistance in Ghana. There is a need for investment in research and monitoring systems especially in the neglected regions of Ghana: Upper West, Brong-Ahafo, Volta and Eastern Regions, where little or no work is conducted to know the extent of antibiotic resistance menace. Additionally, there should be support for higher quality research and incentives for publication in higher impact journals, which will ensure a wider readership. Consequently, this would make researchers visible and complement global efforts to monitor resistance patterns in the country.

This systematic review shared light on an important medical Ghanaian and global issue, however, it has some limitations. The literature search was restricted to only two databases: PubMed and Google Scholar; some journals are not included in those databases and consequently, relevant articles could have been missed through the systematic search methodology used. There is also the possibility that some investigations conducted in Ghana have not been published or yet to be published. There is evidence that a substantial number of researches remain unreported because of the limitations in time and resources as well as researches with findings that do not support the researcher’s hypothesis [87]. The sample size is also a limiting factor with only sixty articles included in this analysis. Twenty of them were not fully available and only the abstracts were screened. Besides, most articles studying human isolates were in the Greater Accra Region. Despite limitations, the findings of this review provide relevant evidence for further actions to address the menace of antibiotic resistance worldwide.

Antibiotic resistance occurs naturally as a survival adaptation of the bacteria, nevertheless, antibiotics misuse in humans and animals accelerates the process. Due to the misuse of antibiotics, governments and health authorities in Ghana and several other African countries must enforce legislation to prevent commercial and indiscriminate use of antibiotics [2]. For example, limiting the sale of antibiotics to pharmacies to ensure that the dispensation of antibiotics is performed by a pharmacist and only if prescribed by a medical doctor. There must be educational programs to sensitize professionals and patients to ensure that antibiotics are only prescribed when needed and that the patients complete the prescribed treatments [88-90]. The enforcement of these measures may look difficult and complicated because of the limiting socio-economic factors. However, in the long term, those measures are cheaper than the higher medical costs and increased mortality as a result of antibiotic resistance [91].

## Conclusion

High resistance rates of microbial isolates from human, animal, food and environment samples have been found in Ghana, especially in *E. coli* (62.2%), *Klebsiella* spp. (60.4%) and *Pseudomonas* spp. (52.1%). Ampicillin, Cefadroxil, Cefotiam, Cloxacillin, Cotrimoxazole, Erythromycin, Penicillin and Trimethoprim showed very high resistance rates (Table 2). However, the data were skewed because some regions of the country have been totally neglected in terms of antibiotic resistance research. There is a need for higher quality research to establish and monitor resistance patterns in Ghana to contribute to the global efforts to curb antibiotic resistance.

### What is known about this topic

- Antibiotic resistance is one of the biggest threats to international health, food security and development nowadays;
- Antibiotic resistance occurs naturally, as a survival adaptation of the bacteria, nevertheless, antibiotics misuse in humans and animals accelerates the process;
- It is crucial to improve surveillance and raise public awareness about this global crisis; enabling public knowledge will increase understanding of proper use of antibiotics and reduce misuse.

### What this study adds

- Compiled available information concerning antibiotic resistance research in Ghana;
- Evidenced the significantly high antibiotic resistance rates in Ghana, especially in *E. coli, Klebsiella* spp. and *Pseudomonas* spp.;
- Identified some gaps about resistance in Ghana: neglected regions and low-quality research.
